# Highly divergent herpesviruses in threatened river dolphins from Brazil

**DOI:** 10.1038/s41598-021-04059-0

**Published:** 2021-12-31

**Authors:** Helena Exposto Novoselecki, José Luiz Catão-Dias, Ana Carolina Ewbank, Pedro Enrique Navas-Suárez, Aricia Duarte-Benvenuto, Henrique Christino Lial, Samira Costa Silva, Angélica María Sánchez-Sarmiento, Waleska Gravena, Vera Maria Ferreira da Silva, Vitor L. Carvalho, Miriam Marmontel, Carolina P. Bertozzi, Vanessa Lanes Ribeiro, Rodrigo del Rio do Valle, Juliana Marigo, Carlos G. das Neves, Fernando Esperón, Carlos Sacristán

**Affiliations:** 1 Laboratory of Wildlife Comparative Pathology, Department of Pathology, School of Veterinary Medicine and Animal Sciences, University of São Paulo, São Paulo, SP 05508-270 Brazil; 2 Instituto Nacional de Pesquisas da Amazônia, Manaus, AM 69067-375 Brazil; 3 Instituto de Saúde e Biotecnologia, Universidade Federal do Amazonas, Coari, AM 69460-000 Brazil; 4 Associação de Pesquisa e Preservação de Ecossistemas Aquáticos, Caucaia, CE 61627-210 Brazil; 5 Instituto de Desenvolvimento Sustentável Mamirauá, Tefé, AM 69553-225 Brazil; 6 Instituto de Biociências, Universidade Estadual Paulista–UNESP, Campus do Litoral Paulista, São Vicente, SP Brazil; 7 Instituto Biopesca, Praia Grande, SP Brazil; 8 Norwegian Veterinary Institute, Arboretveien 57, 1433 Ås, Norway; 9 Veterinary Department, School of Biomedical and Health Sciences, Universidad Europea de Madrid, C/Tajo S/N, Villaviciosa de Odón, 28670 Madrid, Spain

**Keywords:** Microbiology, Molecular biology, Infectious diseases, Viral infection

## Abstract

River dolphins are a highly threatened polyphyletic group comprised of four odontocete families: Iniidae, Pontoporiidae, Lipotidae, and Platanistidae, the first two endemic to South America. To address the knowledge gap regarding infectious agents in this cetacean group, we surveyed the presence of herpesviruses by PCR in skin and/or blood samples of live-captured Amazon (*Inia geoffrensis*, n = 25) and Bolivian (*Inia boliviensis*, n = 22) river dolphins of the Amazon basin and in selected tissue samples of franciscanas (*Pontoporia blainvillei*, n = 27) stranded or bycaught in southeastern Brazil. Additionally, available franciscana tissue samples were examined by histopathology. Herpesvirus DNA was amplified in 13 Bolivian river dolphins (59.1%, 95% CI 38.5–79.6%) and 14 franciscanas (51.9%, 95% CI 33.0–70.7%). All Amazon river dolphins were herpesvirus-negative. Two different herpesviruses were found in Bolivian river dolphins: a previously known gammaherpesvirus detected in blood and/or skin samples of all positive individuals and a novel alphaherpesvirus in the skin of one animal. A new gammaherpesvirus was found in several franciscana samples—the first herpesvirus recorded in Pontoporiidae. Intranuclear inclusion bodies consistent with herpesvirus were observed in the lymph node of one franciscana. The high divergence among the obtained herpesviruses and those previously described can be explained by viral-host coevolution, and by the fact that these populations are fairly isolated.

## Introduction

The polyphyletic group “river dolphins” comprises four different odontocete families: Iniidae, Pontoporiidae, Lipotidae and Platanistidae^1,2^. They are likely relict representatives of originally diverse marine taxa that precede the radiation of the Delphinidae and remained in riverine ecosystems or coastal waters (i.e., Pontoporiidae)^1^. These families are characterized by certain morphological similarities, such as narrow and elongated rostrum, elongated and fused mandibular symphysis, small eyes, flexible neck and broad pectoral fins^1,3^. River dolphins are currently the most threatened group of cetaceans^4^, including the last functionally extinct cetacean species—the baiji (*Lipotes vexillifer*)^5^.

Two families of river dolphins are endemic in South America: Iniidae and Pontoporiidae. According to da Silva^6^ and Hrbek et al.^7^, the Iniidae comprises three species: the Araguaian boto (*Inia araguaiaensis*), the Bolivian river dolphin (*I. boliviensis*), and the Amazon river dolphin or boto (*Inia geoffrensis*). The Iniidae are widely distributed throughout rivers and lakes of the Amazon, Orinoco and Araguaia-Tocantins basins^7,8^, and are strongly dependent on river margins and flooded forests^9,10^. The genus *Inia* is considered Endangered by the IUCN Red List and the Brazilian Ministry of Environment^11,12^ especially due to illegal hunting^11,13^, but also to several other factors such as habitat fragmentation, mining-related pollution (e.g. mercury), deforestation and burning of primary forest, oil exploitation^13–16^, fishing interaction and bycatch^17^, and increased ship traffic^18^.

The Pontoporiidae family comprises only one species—the franciscana (*Pontoporia blainvillei*), also known as toninha or La Plata dolphin. The franciscana is a small cetacean endemic of the southwestern Atlantic Ocean, that inhabits coastal and occasionally estuarine waters from the state of Espírito Santo, southeastern Brazil (18º25′S–30º42′W) to the Chubut province, in the northern Argentinean Patagonia (42º35′S–64º48′W), with some gaps in their distribution^19^. This species is classified as Vulnerable by the IUCN Red List^20^ and as Critically Endangered by the Brazilian Ministry of Environment^12^. The main threats to franciscanas are bycatch in gillnet fisheries^20^, followed by overfishing^21^, chemical pollution^22,23^, and ingestion of plastic debris^24^.

Infectious diseases are recognized as potential threats to species conservation^25^; however, their presence in the genus *Inia* is largely unknown, limited to reports in captive animals^26–30^ and to the description of bacterial and parasitic pneumonias by *Halocercus brasiliensis* in free-ranging Amazon river dolphins^31^. Additionally, we recently described gammaherpesvirus infection in a proliferative skin lesion of a Bolivian river dolphin from the Guaporé River, Brazil^32^. The available literature regarding infectious agents in franciscanas is also extremely limited, represented by the detection of *Staphylococcus* sp., *Micrococcus* sp. *Brucella* sp. and *Enterobacter kobei*^33–35^. Furthermore, gastrointestinal parasites—including the acanthocephalan *Polymorphus* sp. and the trematodes *Hadwenius pontoporiae* and *Synthesium pontoporiae*, among others, have been reported^36–38^.

Herpesviruses (HV) are enveloped double-stranded DNA viruses within the family *Herpesviridae*, subdivided into the subfamilies *Alphaherpesvirinae*, *Betaherpesvirinae* and *Gammaherpesvirinae*^39^. HV is one of the most studied infectious agents in cetaceans, known to host so far alpha- and gammaherpesvirus^32,40,41^. Alphaherpesvirus infections in cetaceans have been associated with cutaneous disease and sometimes fatal localized or systemic infections such as fatal vasculitis and encephalitis^42–45^, but also with subclinical infections^45,46^. In cetaceans, gammaherpesvirus have been detected in asymptomatic infections^46^, and associated with cutaneous^47,48^, but especially with mucosal lesions (mainly genital, oral and oropharyngeal)^42,49–51^, which might be able to impact reproduction in affected populations^52^.

In light of the scarce literature regarding herpesvirus infections in riverine dolphins, our goals were to (1) establish and compare the presence of herpesviruses in threatened wild Amazon river dolphins, Bolivian river dolphins and franciscanas in Brazil and (2) elucidate the phylogeny of the detected herpesviruses.

## Results

### Molecular findings

#### Family Iniidae

Herpesvirus DNA was amplified solely in the Bolivian river dolphins of Guaporé River. Overall, herpesvirus DNA was detected in 59.1% (13/22, 95% confidence interval [CI] 38.5–79.6%) of these individuals by DPOL and/or gB PCR, including cutaneous samples from four animals and blood samples from eleven animals. Two dolphins had concomitant herpesvirus infection in skin and blood (Boto 6-GR and Boto 20-GR). The DPOL gene was amplified in 9.1% (2/22, 95% CI 0.0–21.1%) of skin samples and 55.6% (10/18, 95% CI 32.6–78.5%) of blood samples of these dolphins. Of note, the DPOL sequence amplified from a proliferative skin lesion of Boto 20-GR was previously published by our group^32^. Regarding the gB gene, it was amplified in 9.1% (2/22, 95%CI 0.0–21.1%) of the cutaneous samples—collected from different animals than those with skin samples positive to DPOL gene, and also in 4.5% (1/22, 95% CI 0.0–13.2%) of blood samples. Detailed descriptions of DPOL and gB positive and negative samples are provided in Supplementary Table 1.

Two different DPOL sequences were identified by sequencing; one was present in the proliferative skin lesion of Boto 20-GR (MF999154, previously published) and in blood samples of ten additional dolphins, corresponding to a gammaherpesvirus. The other DPOL sequence was found in a single apparently healthy skin sample of Boto 6-GR, which presented the highest nucleotide identity (62.2%) to the alphaherpesvirus sequences described in two common dolphins (*Delphinus delphis*) from Portugal (MG437208, MG437210, MG437211, MG437212) and one from the Canary Islands, Spain (MN179655). It also presented the highest amino acid identity (61.7%) to alphaherpesviruses from a Cuvier's beaked whale (*Ziphius cavirostris*, ALP00292) of the Mediterranean coast of Spain and a harbor porpoise (*Phocoena phocoena*, AZI95599) from Portugal. Regarding the gB gene, the same sequence was found in the skin samples of two individuals (Boto 14-GR, Boto 17-GR) and in blood sample of another specimen (Boto 20-GR). This gB sequence was most similar (68.7% nt, 72.5% aa) to a gammaherpesvirus sequence detected in a squirrel monkey (*Saimiri sciureus*, AY138584). Overall, gammaherpesvirus sequences were detected in 13.6% (3/22, 95% CI 0.0–28.0%) of the skin samples and 61.1% (11/18, 95% CI 38.6–83.6%) of the blood samples of Bolivian river dolphins, and an alphaherpesvirus was found in 4.5% of the tested skin samples (1/22, 95% CI 0.0–13.2%).

All the samples of Amazon river dolphins from Negro (n = 16, 15 skin samples and one blood sample) and Tapajós (blood samples, n = 9) rivers were negative for herpesvirus on both DPOL and gB PCRs.

#### Family Pontoporiidae

Herpesviral DNA was amplified in 51.9% (14/27, 95% CI 33.0–70.7%) of the tested franciscanas; 28.6% (4/14, 95% CI 4.9–52.2%) of calves, 75% (6/8, 95% CI 45.0–100%) of juveniles and 80% (4/5, 95% CI 44.9–100%) adults. The same DPOL sequence was identified in 14 tissue samples (prescapular lymph node [n = 3], liver [n = 2], mesenteric lymph node [n = 2], mediastinal lymph node [n = 1], unidentified lymph node [n = 1], blood [n = 1], spleen [n = 1], lung [n = 1], testicle [n = 1], skeletal muscle [n = 1] collected from eight animals. It had the highest nucleotide and amino acid identities (70.1% and 70.9%, respectively) to the gammaherpesvirus sequence KP995688, found in a common minke whale (*Balaenoptera acutorostrata*) from Spain. Regarding gB, the same sequence was found in 24 samples (lung [n = 4], blood [n = 3]), prescapular lymph node [n = 3], mesenteric lymph node [n = 3], spleen [n = 2], mediastinal lymph node [n = 1], thymus [n = 1], unidentified lymph node [n = 1], heart [n = 1], liver [n = 1], skin [n = 1], spinal cord [n = 1], skeletal muscle [n = 1], and testicle [n = 1]) of 12 franciscanas, and had the highest nucleotide (68.9%) and amino acid identities (72.7%) with gammaherpesvirus sequences found in a common vampire bat (*Desmodus rotundus*, MN850469) and squirrel monkey (*Saimiri sciureus*, AY138584), respectively. Detailed descriptions of DPOL and gB positive and negative samples are provided in Supplementary Table 2.

The DPOL phylogenetic analysis revealed that the sequences from Bolivian river dolphins and franciscanas were not grouped into any of the alpha- or gammaherpesvirus genera of mammals recognized by the ICTV.

On the DPOL phylogram, the sequence obtained from the Bolivian river dolphin Boto 6-GR did not cluster with other alphaherpesviruses. In contrast, the remaining sequences from Bolivian river dolphins and franciscanas grouped with the gammaherpesvirus sequences included in the alignment. The gB phylogenetic analysis revealed that the gammaherpesvirus sequences from Bolivian river dolphins and franciscanas clustered with other gammaherpesvirus of the genus *Rhadinovirus*.

Representative DPOL sequences obtained in this study were submitted to GenBank under accession numbers MZ223442 (gammaherpesvirus obtained from the skin of the Bolivian river dolphin Boto 1-GR), MZ223443 (alphaherpesvirus obtained from the skin of the Bolivian river dolphin Boto 6-GR) and MZ223444 (gammaherpesvirus from lung, skeletal muscle and prescapular lymph node of franciscana MM568). Representative gB sequences were submitted under accession numbers MZ209258 (gammaherpesvirus obtained from the skin of the Bolivian river dolphin Boto 14-GR) and MZ209259 (gammaherpesvirus identified in lung, skeletal muscle, prescapular lymph node, thymus and skin samples of franciscana MM568). Unfortunately, it was not possible to obtain the complete HV genome sequence in any of the HV-positive samples tested by metagenomics (one skin lesion and four blood samples of five Bolivian river dolphins and prescapular lymph node, skeletal muscle, thymus and lung of the franciscana MM568).

### Histopathological findings

**Figure 1:**
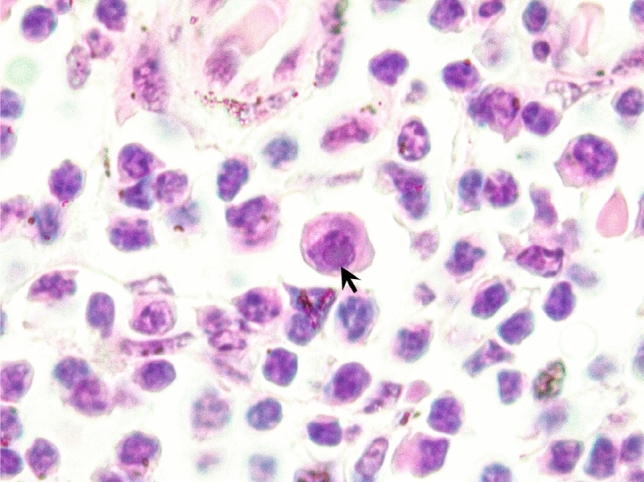
Lymph node of a franciscana (*Pontoporia blainvillei*, case MM568). Note the presence of a basophilic intranuclear inclusion body in a macrophage (black arrow).

There were no available formalin-fixed paraffin embedded skin tissue samples from the novel HV-positive *Inia*. Regarding the HV-positive franciscanas, one of them (MM568) presented intranuclear inclusion bodies consistent with typical herpesviral inclusions in lymph nodes (Fig. 1). No other lesions consistent with herpesvirus infection were observed. The microscopic findings of HV-positive franciscanas are recorded in Supplementary Table 3.

## Discussion

This study reports the presence of herpesviruses in Bolivian river dolphins and franciscanas in Brazil. All Amazon river dolphins were negative. Gammaherpesvirus sequences were detected in 64.7% of the blood and 13.6% of the skin samples of Bolivian river dolphins, and an alphaherpesvirus was found solely in the skin sample of one individual. Additionally, a hereinto unknown gammaherpesvirus sequence was found in 51.9% of the tested franciscanas. In South American cetaceans, the presence of herpesviruses had been reported in two Guiana dolphin (*Sotalia guianensis*), a Bolivian river dolphin (included in this study, Boto 20-GR), a dwarf sperm whale (*Kogia sima*), and an Atlantic spotted dolphin (*Stenella frontalis*)^32,53^. To the authors’ knowledge, this is the first report of an alphaherpesvirus in a Bolivian river dolphin worldwide, and also of herpesvirus in a franciscana. One Bolivian river dolphin (Boto 6-GR) had a gammaherpesvirus and an alphaherpesvirus. The presence of different herpesviral sequences in the same individual has been previously described in other cetacean species, as in Atlantic bottlenose dolphin (*Tursiops truncatus*)^42^, striped dolphin (*Stenella coeruleoalba*)^46,54,55^, dwarf sperm whale^32^ and common dolphin (*Delphinus delphis*)^56^.

High gammaherpesvirus prevalence was observed in the studied Bolivian river dolphins and franciscanas, indicating that these species are likely the natural host of the detected viruses^57,58^. Moreover, in the case of franciscanas, the same agent appeared in different years (2011–2014, and 2019–2020), reinforcing our hypothesis. The three herpesviruses found in river dolphins (Bolivian river dolphins and franciscanas) were highly divergent from the closest sequences available in GenBank. The gammaherpesvirus found in Bolivian river dolphins was previously tentatively denominated *Iniid herpesvirus 1*^32^. According to the current HV nomenclature used by the ICTV^39^, we propose renaming the virus as *Iniid gammaherpesvirus 1*. The novel unique sequences found in this study likely correspond to novel herpesvirus species, herein proposed as *Iniid alphaherpesvirus 1* for the novel alphaherpesvirus found in a Bolivian river dolphin skin sample, and *Pontoporiid gammaherpesvirus 1* for the gammaherpesvirus discovered in franciscanas. The large differences between the herpesviruses found in river dolphins from South America and those previously described in marine cetaceans can be explained by the co-evolution of the viruses with their hosts, as described for other members of the family *Herpesviridae*^59^. According to McGowen et al.^60^, the families Iniidae and Pontoporiidae, as well as Lipotidae, separated from the other odontocete cetaceans around the Late Oligocene, between 23.03 and 24.92 million years ago. We hypothesize the herpesviruses detected in the South American river dolphin co-evolved with their host during millions of years, which likely explain the large differences observed in well-preserved herpesviral genes (DNA polymerase and glycoprotein B) when compared to those detected in other cetacean families. Another possibility would be the cross-species transmission from unidentified host species. Further studies are necessary to explore these hypotheses.

The absence of herpesvirus detection in the Amazon river dolphins was unexpected, especially from those of Tapajós River, which inhabit an area intensely affected by anthropogenic activities^61^. Specifically, the Tapajós River Basin hosts the largest small-scale gold mining (“garimpo” in Portuguese) district in the world^62^, an activity known for using large amounts of mercury, water siltation, and habitat fragmentation and degradation^63–65^. In mammals, mercury intoxication causes neurological and reproductive disorders, and immunosuppression^66–68^. High total mercury loads (from 0.61 to 2.3 mg/kg wet weight) were recently described in muscle samples of eight of the Amazon river dolphins from the Tapajós River^69^, also analyzed in the present study. Five Amazon river dolphins from the Tapajós River were in poor nutritional condition, with three individuals presenting loss of nucal fat and one of them also presenting marked scapula and rib cage. By contrast, all the Amazon river dolphins from Negro River and Bolivian river dolphins from Guaporé River apparently were in good nutritional condition. It is well known that immunosuppression, as a result of concurrent disease, stress and reproductive effort, may reactivate latent herpesvirus infections^70^, leading to periodic or continuous shedding of infectious virions and destruction of the infected cell^59,70,71^, consequently increasing the likelihood of their detection. The absence of herpesvirus detection in the Amazon river dolphins may be explained by (1) the low number of tested samples from Amazon river dolphins captured in Tapajós River (2) apparent good health status of Amazon river dolphins from Negro River, (3) a lack of sensitivity of the employed methods to detect another divergent herpesvirus infecting Amazon river dolphins or (4) differences in the evolutionary history of Amazon and Bolivian river dolphins and their herpesviruses, once herpesviruses and their natural hosts are intimately related, usually sharing a common evolutionary history and mutual adaptation^59^. Future studies with a larger sample size are necessary to clarify the difference in herpesvirus prevalence between Amazon and Bolivian river dolphins.

Regarding the potential pathogenicity of the riverine dolphin herpesviruses, the gammaherpesvirus detected in several Bolivian river dolphins was previously identified in a proliferative skin lesion of one individual from the same population (Boto 20-GR), and might be associated with its etiology^32^. Additionally, an alphaherpesvirus was amplified from a cutaneous sample of another Bolivian river dolphin (Boto 6-GR). Unfortunately, only the proliferative cutaneous lesion of Boto 20-GR was histologically evaluated, due to the lack of available fixed samples from the remaining HV-positive individuals. No gross cutaneous lesions were observed in other HV-positive Bolivian river dolphins aside from Boto 20-GR. In franciscanas, no evidences of herpesvirus infection were found aside from amphophilic intranuclear inclusion bodies in one individual. The lack of associated lesions was also observed in 12 herpesvirus-positive striped dolphins (*Stenella coeruleoalba*) from Italy^72^. It is important to remark that many herpesviruses only cause mild disease in their natural host^59^, and the absence of HV-associated lesions in the examined franciscanas with gammaherpesvirus and in most of the alpha- and gammaherpesvirus-positive Bolivian river dolphins may be examples of the low pathogenicity of some HVs. Nevertheless, the *Iniid alphaherpesvirus 1* and *Pontoporiid gammaherpesvirus 1* pathogenicity should be further investigated.

The main pathological findings observed in the herpesvirus-positive franciscanas were linear cutaneous lacerations and imprints, multi-organ congestion, pulmonary edema and myocyte and cardiomyocyte degeneration and necrosis. In cetaceans, although non-specific, these lesions are usually related to peracute underwater entrapment syndrome associated with bycatch^73–75^. Cardiomyocyte and myocyte hypereosinophilia have been also described in human drowning cases^76^. The pathological findings are in agreement with the franciscanas’ records since several of these animals were directly recovered entangled in fishing nets and handed by the fisherman to the researchers. Bycatch is considered the main threat affecting franciscana’s conservation, especially entanglement in gillnets^20^. The population recovery from the current high levels of bycatch across this species range is limited due to franciscana’s short life span and low reproductive potential^77,78^. Although several studies tested alternative approaches and techniques with artisanal fisheries, no solutions have significantly reduced the mortality rates associated with this impact^79,80^. The high occurrence of fisheries interaction observed in our study reinforces the need to develop management strategies to reduce the impact of incidental captures of this species in the studied region.

## Conclusion

Herein we described the presence of two herpesviruses in Bolivian river dolphins: a previously known gammaherpesvirus detected in blood and skin samples, and a novel alphaherpesvirus species present in a skin sample. A new gammaherpesvirus species was also found in several tissue samples from franciscanas, the first herpesvirus ever recorded in the family Pontoporiidae. Based on the high gammaherpesvirus prevalence observed in Bolivian river dolphin (59.1%, 13/22) and franciscana (51.9%, 14/27) and the absence of associated lesions in most of the animals (with the exception of the skin lesion observed in Boto 20-GR), these species are likely their natural hosts. Of note, the obtained sequences were highly divergent when compared to other herpesviruses available on public databases, which could be explained by viral-host coevolution—once these riverine species diverged from the other odontocete cetaceans millions of years ago, and by the fact that these populations are fairly isolated to this date. Future studies are warranted to elucidate the pathological potential and impact of these novel viruses in highly threatened riverine cetaceans.

## Methods

### Samples

#### Family Iniidae

**Figure 2:**
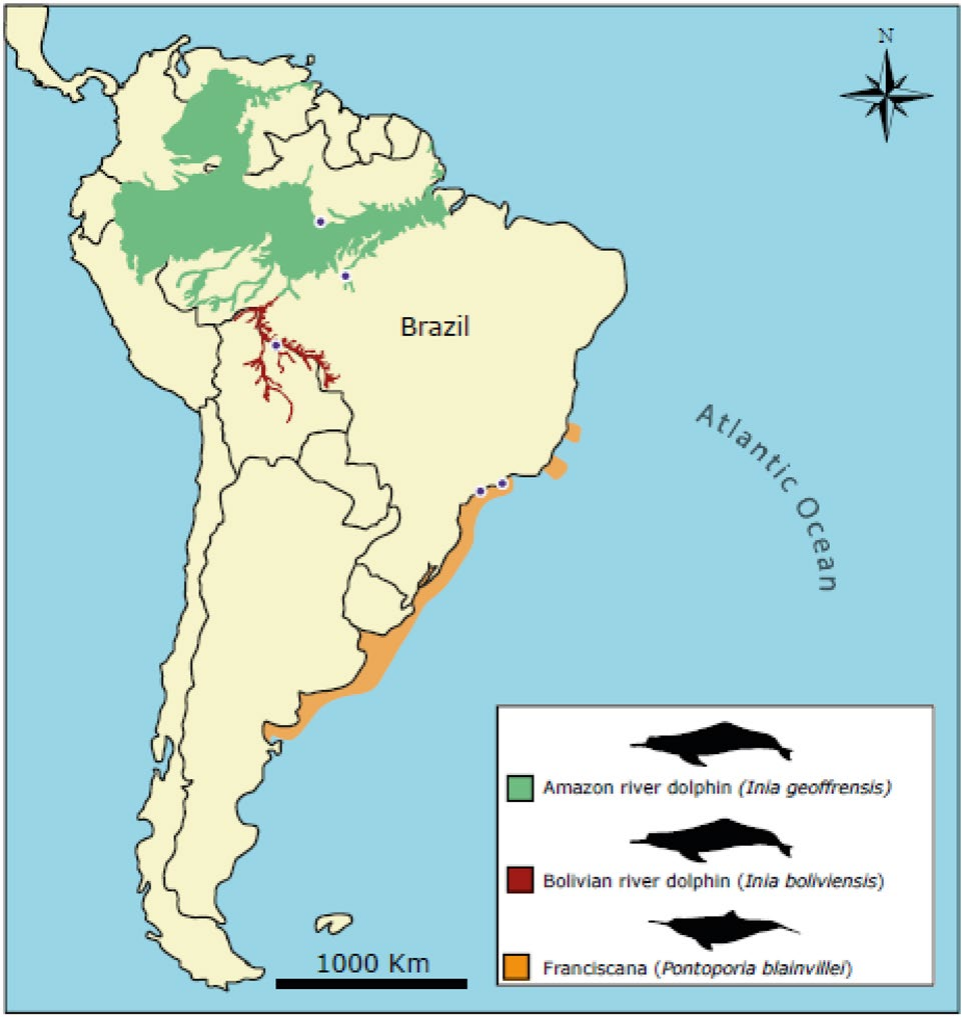
Location of the Bolivian river dolphins (*Inia boliviensis*), Amazon river dolphins (*Inia geoffrensis*) and franciscanas (*Pontoporia blainvillei*) tested in the study. The distribution of the species was based on the platform Botos Amazônicos for the genus Inia and on Zerbini et al. (2018) for Franciscanas. The collection sites are marked with blue.

Samples of 47 *Inia* (39 males and eight females) were collected in expeditions conducted in the Brazilian Amazon basin in 2015 and 2017 (Fig. 2). In February and December 2015, respectively, 16 Amazon river dolphins were captured in Negro River (03°17′ 12.96″S, 52°15′12.16″W, and 03°04′10″S 60°18′41″W, Amazonas state). In September of the same year, 22 Bolivian river dolphins were captured in Guaporé River (12°27′37.87″S, 64°17′ 20.95″W–S 12°29′32.8″S, 64°03′26.1″, Rondônia state). Finally, in October 2017, nine Amazon river dolphins were sampled in a third expedition, to the Tapajós River (07º20′35.79″S, 58º8′53.78″W, Pará state). The *Inia* captures followed the methodology described by da Silva and Martin^81^, being immediately released after sampling. Nutritional condition was estimated by a veterinarian during physical examination. Detailed information about the sex and age class of animals is displayed in Table 1.

**Table 1:** Description of the analyzed individuals of the families Iniidae and Pontoporiidae: species, location (AM: Amazon state, RO: Rondônia state, PA: Pará state, SP: São Paulo state), date of capture (*Inia* spp.) or necropsy (*Pontoporia blainvillei*), number of evaluated specimens (n), sex (M: male, F: female), age class (C: calf, J: juvenile, A: adult, U: unknown), and number of samples (skin, blood and other tissue samples).

	Family	Species	Location	Date of capture (*Inia* spp.)/necropsy (*Pontoporia blainvillei*)	n	Sex		Age class			Number of samples^b^		
						M	F	C^a^	J	A	Skin	Blood	Other tissues
Odontoceti	Iniidae	Amazon river dolphin (*Inia geoffrensis*)	Negro River (AM)	February and December 2015	16	16	–	2	6	8	15	1	–
		Bolivian river dolphin (*Inia boliviensis*)	Guaporé River (RO)	September 2015	22	16	6	2	6	14	22 (**4**)	18 (**11**)	–
		Amazon river dolphin (*Inia geoffrensis*)	Tapajós River (PA)	October 2017	9	7	2	1	2	6	–	9	–
	Pontoporiidae	Franciscana (*Pontoporia blainvillei*)	SP	2001–2020	27	17	10	14	8	5	2 (**1**)	11 (**3**)	78 (**22**)
		Total			74	56	18	18	20	27	39 (**5**)	39 (**14**)	78 (**22**)

Values in bold and parentheses correspond to the number of positive samples.

^a^One of the individuals included in this group was a fetus.

^b^The number of positive samples is specified in the parentheses.

Overall, 37 skin samples and 28 blood samples were obtained from Amazon river dolphins captured at Negro River (15 skin samples and 1 blood sample), Bolivian river dolphins from Guaporé River (22 skin samples and 18 blood samples) and Amazon river dolphins from Tapajós River (9 blood samples). Blood samples were collected from the ventral caudal peduncle and placed in Vacutainer tubes with EDTA. Biopsied skin samples were collected from the caudal and dorsal fins (n = 36) or cutaneous lesions (n = 1). Blood and skin samples were kept at − 80 °C until analysis.

#### Family Pontoporiidae

Overall, 91 tissue samples (adrenal gland, blood, brain, heart, kidney, liver, lung, lymph nodes, skeletal muscle, skin, spinal cord, spleen, testicle, thymus, and uterus) of 27 franciscanas stranded or bycaught in the coast of southeastern Brazil (São Paulo state, Fig. 2) between 2013 and 2020 were analyzed. The animals were necropsied following standard procedures^82^. The age class was established according to total body length, as described by Rosas and Monteiro-Filho^83^, using the values of the Franciscana management area II (São Paulo, Paraná and Santa Catarina states). In three franciscanas the exact age was determined through the histology of the teeth^84,85^. Selected tissue samples were fixed in 10% formalin at room temperature. An additional set of samples was frozen at − 20 °C or − 80 °C until analysis. Additional data regarding the franciscanas are displayed in Table 1.

### Molecular assays

Total DNA was extracted from frozen samples using the kit DNA Blood and Tissue (Qiagen, Germany). The panPCR nested protocol described by VanDevanter et al.^86^, able to amplify alpha-, beta- and gammaherpesviruses, was selected to partially amplify the herpesvirus DNA polymerase gene (DPOL). The PCR protocol described by Ehlers et al.^87^ using the primer set GH1 to amplify the glycoprotein B (gB) gene of gammaherpesviruses was also performed in all available samples.

**Figure 3:**
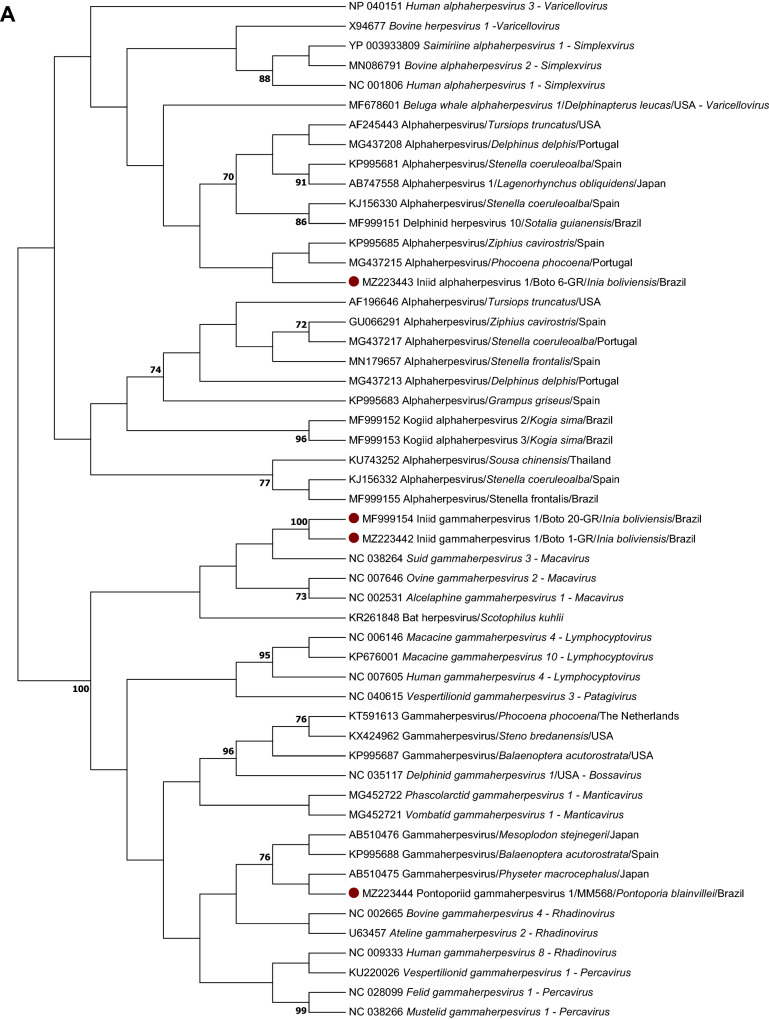

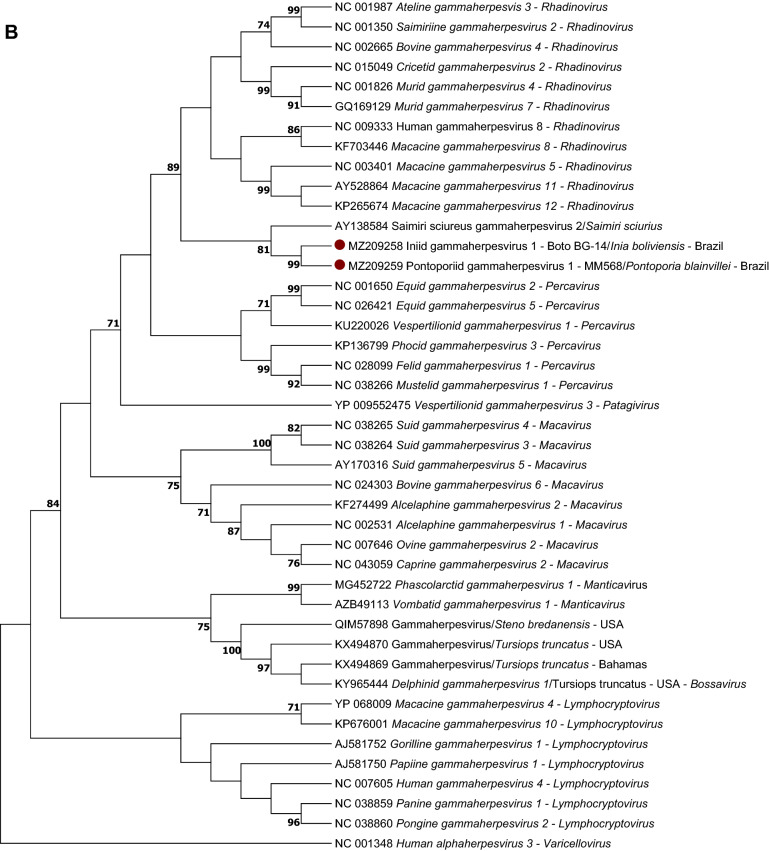
(**A**) Maximum likelihood phylogram based on the JTT matrix-based model of the deduced amino acid DNA polymerase sequences: (i) obtained in this study (red dots), (ii) the most similar sequence form GenBank, (iii) alpha- and gammaherpesvirus sequences obtained from other cetacean species, (iv) accepted alpha- and gammaherpesvirus species recognized by the International Committee on Taxonomy of Viruses. Maximum likelihood phylogram based on the JTT matrix-based model of the deduced amino acid glycoprotein B sequences (i) obtained in this study (red dots), (ii) the most similar sequence form GenBank, (iii) gammaherpesvirus sequences obtained from other cetacean species, (iv) gammaherpesvirus species recognized by the International Committee on Taxonomy of Viruses. Human alphaherpesvirus 3 was selected as outgroup for glycoprotein B phylogram. The reliability of the tree was tested by bootstrap analysis with 1000 replicates, and those bootstrap values lower than 70 were omitted.

Two skin samples (Boto 17-GR and Boto 20-GR) and one blood sample (Boto 20-GR) of Bolivian river dolphins captured at Guaporé River, as well as a skin sample from one Amazon river dolphin of Negro river (Boto 12-NR), were previously tested by DPOL PCR^32^. The skin sample of Boto 20-GR was also tested for HV gB^32^. A Magellanic penguin alphaherpesvirus and a fur seal gammaherpesvirus were selected as positive controls^88,89^. DPEC water was used as no template control. Positive amplicons were purified using ExoSap-IT (USB Corporation, Ohio, USA) and directly sequenced by Sanger using the ABI 3730 DNA Analyser. The obtained sequences were aligned in MEGA Software 7.0 by Muscle to obtain the consensus sequences^90^. After editing out the primers, these consensus sequences were compared to those available at GenBank by Blast search, and p-distance was calculated to obtain the percentage of identity ([1 – p-distance) * 100]). Finally, DPOL and gB Maximum likelihood phylograms based on the deduced amino acid sequences of the herpesviruses found in this study, representative herpesvirus species recognized by the International Committee on Viral Taxonomy (ICTV) and herpesvirus previously identified in cetaceans were constructed using MEGA 7.0 (Fig. 3) with a bootstrap value of 1000 replications. All bootstrap frequency values less than 70 were omitted. The *Human alphaherpesvirus 3* was selected as outgroup for gB phylogram.

Tissue samples from selected HV-positive animal were submitted to complete sequencing at the University of Florida.

### Histopathological examination

Available formalin-fixed samples from HV-positive cases were embedded in paraffin wax, processed as routine, sectioned at 5 μm and stained with hematoxylin and eosin for light microscopic examination.

### Ethical standards

The Amazon river dolphins from the Tapajós river were sampled as part of the South American River Dolphin (SARDI) integrated strategy for the conservation of South American river dolphins, funded by WWF. The Amazon river dolphins from Negro river were sampled as part of Projeto Mamíferos Aquáticos da Amazonia and sponsored by Ampa/Petrobras Socioambiental. All study samples were collected in full compliance with specific federal permits issued by the Brazilian Ministry of Environment (MMA) and the Chico Mendes Institute for Biodiversity Conservation (ICMBio), and approved by the Biodiversity Information and Authorization System (SISBIO 31226-1/2, 47780-4, 49597-1, 60171-1 and 72608-1), ABIO Nº 1169/2019 and ICMBio/MMA (13157). Three fransciscanas were collected as part of the Santos Basin Beach Monitoring Project (Projeto de Monitoramento de Praias da Bacia de Santos—PMP-BS), licensed by the Brazilian Institute of the Environment and Renewable Natural Resources (IBAMA) of the Brazilian Ministry of Environment (ABIO Nº 1169/2019). All procedures were performed in accordance with the Ethical Committee in Animal Research of the School of Veterinary Medicine and Animal Sciences, University of São Paulo or the Ethical Committee of Animal Use of the National Institute of the Amazon Research, the latter responsible for the approval of the protocol for handling and removing of small samples of epithelial tissue and blood from the botos (Process numbers CEUA 7151291019 and INPA 01/2013, respectively). ARRIVE guidelines: not applicable.

## Supplementary Information

Supplementary Information.

## Data Availability

All data generated or analyzed during this study are included in this published article.
