# Supplementary material for: Highly divergent herpesviruses in threatened river dolphins from Brazil
Source: Sci Rep. 2021 Dec 31;11:24528. doi: 10.1038/s41598-021-04059-0 (PMC8720088; doi:10.1038/s41598-021-04059-0)
Supplement: Supplementary file 1 — Supplementary Information. [file 41598_2021_4059_MOESM1_ESM.docx]

**SUPPLEMENTARY TABLES**

**Supplementary Table 1**. Herpesvirus DNA polymerase and glycoprotein B PCR results on blood and skin samples of Bolivian river dolphins (*Inia boliviensis*) from the Guapore River. All the PCR results were confirmed by sequencing. All the obtained sequences correspond to gammaherpesvirus except the alphaherpesvirus obtained from the skin of Boto 6-GR. Data about sex (M: male, F: female), age class (C: calf, J: juvenile, A: adult) and total body length (TBL, in centimeters) are also provided. Herpesvirus PCR-positive cases are typed in red.

| **ID** | **Species** | **Sex** | **Age class** | **TBL (cm)** | **SKIN** | | **BLOOD** | |
| --- | --- | --- | --- | --- | --- | --- | --- | --- |
|  |  |  |  |  | **DPOL** | **gB** | **DPOL** | **gB** |
| **Boto 1-GR** | *I. boliviensis* | M | A | 210 | 0 | 0 | **1** | 0 |
| **Boto 2-GR** | *I. boliviensis* | M | A | 197 | 0 | 0 | **1** | 0 |
| Boto 3-GR | *I. boliviensis* | M | C | 100 | 0 | 0 | 0 | 0 |
| **Boto 4-GR** | *I. boliviensis* | F | A | 199 | 0 | 0 | **1** | 0 |
| Boto 5-GR | *I. boliviensis* | M | A | 209 | 0 | 0 | - | - |
| **Boto 6-GR** | *I. boliviensis* | F | J | 157 | **1** | 0 | **1** | 0 |
| **Boto 7-GR** | *I. boliviensis* | M | J | 169 | 0 | 0 | **1** | 0 |
| **Boto 8-GR** | *I. boliviensis* | F | A | 215 | 0 | 0 | **1** | 0 |
| Boto 9-GR | *I. boliviensis* | M | C | 133 | 0 | 0 | 0 | 0 |
| Boto 10-GR | *I. boliviensis* | F | A | 205 | 0 | 0 | 0 | 0 |
| Boto 11-GR | *I. boliviensis* | M | A | 193 | 0 | 0 | - | - |
| Boto 12-GR | *I. boliviensis* | M | A | 205 | 0 | 0 | 0 | 0 |
| **Boto 13-GR** | *I. boliviensis* | M | A | 200 | 0 | 0 | **1** | 0 |
| **Boto 14-GR** | *I. boliviensis* | M | J | 177 | 0 | **1** | - | - |
| Boto 15-GR | *I. boliviensis* | F | J | 147 | 0 | 0 | - | - |
| Boto 16-GR | *I. boliviensis* | M | J | 174 | 0 | 0 | 0 | 0 |
| **Boto 17-GR** | *I. boliviensis* | M | J | 186 | 0 | **1** | 0 | 0 |
| **Boto 18-GR** | *I. boliviensis* | M | A | 222 | 0 | 0 | **1** | 0 |
| **Boto 19-GR** | *I. boliviensis* | F | A | 194 | 0 | 0 | **1** | 0 |
| **Boto 20-GR** | *I. boliviensis* | M | A | 198 | **1*** | 0 | 0 | **1** |
| **Boto 21-GR** | *I. boliviensis* | M | A | 210 | 0 | 0 | **1** | 0 |
| Boto 22-GR | *I. boliviensis* | M | A | 211 | 0 | 0 | 0 | 0 |
| ***Overall*** |  |  |  |  | ***2*** | ***2*** | ***10*** | ***1*** |

*The DNA polymerase sequence of Boto 20-GR was previously identified in a proliferative skin lesion by Sacristán et al. 2019.

**Supplementary Table 2**. DNA polymerase and glycoprotein B PCR results on the tested samples of franciscanas (*Pontoporia blainvillei*) from São Paulo state. All the PCR results were confirmed by sequencing. All the obtained sequences correspond to gammaherpesvirus. Data about sex (M: male, F: female), age class (C: calf, J: juvenile, A: adult), total body length (TBL, in centimeters) and data of necropsy are also provided.

| **ID** | **Sex** | **Age class** | | **TBL (cm)** | **Date of Necropsy** | **Tissue** | **PCR** | |
| --- | --- | --- | --- | --- | --- | --- | --- | --- |
|  |  |  |  |  |  |  | **DPOL** | **gB** |
| MM 165 | M | C | | 69 | Nov 2001 | Liver | 0 | 0 |
| MM 172 | M | C | | 57.5 | Dec 2002 | Liver | 0 | 0 |
|  |  |  |  |  |  | Lung | 0 | 0 |
| **MM 332** | M | C | | 91 | Jun 2011 | Spleen | 0 | 0 |
|  |  |  |  |  |  | Blood | 0 | **1** |
|  |  |  |  |  |  | Lung | 0 | **1** |
|  |  |  |  |  |  | Mesenteric lymph node | 0 | 0 |
| **MM 333** | F | A* | | 141 | Jun 2011 | Blood | 0 | 0 |
|  |  |  |  |  |  | Lung | 0 | 0 |
|  |  |  |  |  |  | Spleen | 0 | **1** |
|  |  |  |  |  |  | Mesenteric lymph node | 0 | **1** |
| MM 334 | F | C (fetus) | | 26.5 | Jun 2011 | Lung | 0 | 0 |
| MM 349 | M | C | | 82 | Jul 2011 | Lung | 0 | 0 |
|  |  |  |  |  |  | Blood | 0 | 0 |
|  |  |  |  |  |  | Mediastinal lymph node | 0 | 0 |
|  |  |  |  |  |  | Prescapular lymph node | 0 | 0 |
|  |  |  |  |  |  | Mesenteric lymph node | 0 | 0 |
|  |  |  |  |  |  | Spleen | 0 | 0 |
| MM 350 | M | J | | 111 | Aug 2002 | Lung | 0 | 0 |
| **MM 395** | M | J | | 106 | Oct 2001 | Liver | 0 | 0 |
|  |  |  |  |  |  | Lung | 0 | **1** |
| MM 404 | M | C | | 87 | Oct 2011 | Spleen | 0 | 0 |
|  |  |  |  |  |  | Blood | 0 | 0 |
| **MM 405** | F | J | | 97 | Oct 2011 | Blood | **1** | **1** |
|  |  |  |  |  |  | Lung | 0 | 0 |
| MM 406 | M | J | | 114 | Oct 2011 | Blood | 0 | 0 |
| MM 407 | M | C | | 85 | Oct 2011 | Blood | 0 | 0 |
| MM 419 | F | C | | 62 | Nov 2011 | Brain | 0 | 0 |
|  |  |  |  |  |  | Mesenteric lymph node | 0 | 0 |
|  |  |  |  |  |  | Blood | 0 | 0 |
|  |  |  |  |  |  | Liver | 0 | 0 |
|  |  |  |  |  |  | Lung | 0 | 0 |
| **MM 420** | M | C | 66 | | Nov 2011 | Liver | 0 | 0 |
|  |  |  |  |  |  | Lung | 0 | 0 |
|  |  |  |  |  |  | Brain | 0 | 0 |
|  |  |  |  |  |  | Heart | 0 | **1** |
|  |  |  |  |  |  | Mesenteric lymph node | 0 | 0 |
|  |  |  |  |  |  | Adrenal gland | 0 | 0 |
| **MM 421** | F | A | 139 | | Jan 2012 | Blood | 0 | **1** |
| MM 486 | F | C | - | | - | Brain | 0 | 0 |
|  |  |  |  |  |  | Lung | 0 | 0 |
|  |  |  |  |  |  | Spinal cord | 0 | 0 |
| **MM 548** | M | J | 103 | | Oct 2013 | Blood | 0 | 0 |
|  |  |  |  |  |  | Central nervous system | 0 | 0 |
|  |  |  |  |  |  | Lung | 0 | 0 |
|  |  |  |  |  |  | Rectal lymph node | 0 | 0 |
|  |  |  |  |  |  | Mediastinal lymph node | 0 | 0 |
|  |  |  |  |  |  | Prescapular lymph node | **1** | **1** |
|  |  |  |  |  |  | Mesenteric lymph node | 0 | 0 |
| **MM 549** | M | J | 113 | | Nov 2013 | Brain | 0 | 0 |
|  |  |  |  |  |  | Lung | 0 | 0 |
|  |  |  |  |  |  | Spinal cord | 0 | **1** |
|  |  |  |  |  |  | Mesenteric lymph node | 0 | **1** |
|  |  |  |  |  |  | Testicle | 0 | 0 |
| **MM 550** | M | J | 104 | | Nov 2013 | Prescapular lymph node | 0 | 0 |
|  |  |  |  |  |  | Mesenteric lymph node | **1** | 0 |
|  |  |  |  |  |  | Lung | 0 | **1** |
|  |  |  |  |  |  | Testicle | **1** | **1** |
| MM 565 | M | A | 117 | | Jan 2014 | Brain | 0 | 0 |
|  |  |  |  |  |  | Liver | 0 | 0 |
| **MM 566** | M | J | 110 | | Jan 2014 | Brain | 0 | 0 |
|  |  |  |  |  |  | Prescapular lymph node | **1** | **1** |
|  |  |  |  |  |  | Mediastinal lymph node | **1** | **1** |
|  |  |  |  |  |  | Mesenteric lymph node | **1** | **1** |
| MM 567 | M | C | 82 | | Jan 2014 | Brain | 0 | 0 |
|  |  |  |  |  |  | Lung | 0 | 0 |
|  |  |  |  |  |  | Mesenteric lymph node | 0 | 0 |
| **MM 568** | M | C | 81.5 | | Jan 2014 | Lung | **1** | **1** |
|  |  |  |  |  |  | Skeletal muscle | **1** | **1** |
|  |  |  |  |  |  | Prescapular lymph node | **1** | **1** |
|  |  |  |  |  |  | Mesenteric lymph node | 0 | 0 |
|  |  |  |  |  |  | Heart | 0 | 0 |
|  |  |  |  |  |  | Thymus | 0 | **1** |
|  |  |  |  |  |  | Kidney | 0 | 0 |
|  |  |  |  |  |  | Adrenal gland | 0 | 0 |
|  |  |  |  |  |  | Skin | 0 | **1** |
| MM 569 | F | C | 71 | | Mar 2014 | Blood | 0 | 0 |
|  |  |  |  |  |  | Lung | 0 | 0 |
|  |  |  |  |  |  | Prescapular lymph node | 0 | 0 |
|  |  |  |  |  |  | Brain | 0 | 0 |
|  |  |  |  |  |  | Mesenteric lymph node | 0 | 0 |
|  |  |  |  |  |  | Mediastinal lymph node | 0 | 0 |
| **ii151268** | F | C | 58.4 | | Nov 2019 | Liver | **1** | 0 |
| **ii166901** | F | A | 130.4 | | Mar 2020 | Liver | **1** | **1** |
| **ii169562** | F | A | - | | Jul 2020 | Lymph node | **1** | **1** |
|  |  |  |  |  |  | Spleen | **1** | **1** |
|  |  |  |  |  |  | Lung | 0 | 0 |
|  |  |  |  |  |  | Liver | 0 | 0 |
|  |  |  |  |  |  | Skin lesion | 0 | 0 |
|  |  |  |  |  |  | Central nervous system | 0 | 0 |
|  |  |  |  |  |  | Uterus | 0 | 0 |
| ***Overall*** |  |  |  | |  |  | **14** | **24** |

*Case MM333 is the mother of fetus MM334.

**Supplementary Table 3**. Anatomopathological findings of the herpesvirus-positive franciscanas (*Pontoporia blainvillei*).

| **Id** | **Gross findings** | **Microscopic findings** |
| --- | --- | --- |
| MM332 | Presence of vibrissae hair; skin lesions in the rostrum, dorsal fin and next to the right pectoral fin compatible with gillnet entanglement; moderate meningeal congestion, pulmonary edema, liquid in the abdominal cavity, moderate hepatomegaly. | **Adrenal gland:** marked, focally extensive, pericapsular hemorrhage.  **Cerebrum:** mild, multifocal, acute hemorrhage in the neuroparenchyma.  **Cerebellum:** mild to moderate, focal, leptomeningeal hemorrhage; mild, multifocal, degeneration and necrosis of Purkinje cells.  **Kidney:** mild/moderate, multifocal, corticomedullar congestion; mild, multifocal, tubular degeneration; mild, multifocal, medullar tubular mineralization.  **Liver:** mild, multifocal, hydropic degeneration of hepatocytes; mild, multifocal, dissociation of hepatic trabecula; mild, multifocal, sinusoidal infiltration of mastocytes; mild, multifocal, hepatocellular single cell necrosis/apoptosis; mild, multifocal, microgoticular steatosis.  **Lungs:** mild, multifocal, acute alveolar hemorrhage; mild, multifocal, alveolar histiocytosis; multiple foci of foreign material (agonic aspiration), mild/moderate, focally extensive, subpleural interstitial edema.  **Lymph node:** mild, multifocal, neutrophilic lymphadenitis; mild, multifocal, infiltration of mastocytes.  **Skeletal muscle:** mild, multifocal, myofiber degeneration and necrosis.  **Spleen:** moderate white pulp hyperplasia; mild, multifocal, histiocytosis; rare eosinophilic infiltrates in the red pulp.  **Tonsil:** mild follicular hyperplasia; minimal, focal necrotizing tonsillitis.  **Eyes, heart, lymph node, pancreas, skin, small and large intestine, spinal cord, testicle, thymus, and trachea:** NSFO. |
| MM333 | Skin lesions compatible with gillnet entanglement in the rostrum, thoracic region, pectoral and caudal fin; mild meningeal congestion; multifocal whitish circular areas with irregular boards in the lungs; petechial hemorrhages in the pleura, presence of serum-sanguinolent liquid in the abdominal cavity and pericardium, presence of a fetus. | **Kidney:** marked; focally extensive; subcapsular acute hemorrhage; mild to moderate, multifocal, corticomedullar congestion; mild, multifocal, medullar tubular mineralization.  **Liver:** mild, multifocal, dissociation of hepatic trabecula; mild, multifocal, hepatocellular single cell necrosis/apoptosis.  **Lungs:** marked, focally extensive, septal congestion; moderate, focally extensive, alveolar edema; mild, multifocal, alveolar infiltrates of Mast cells.  **Skeletal muscle:** mild, multifocal, myofiber degeneration and necrosis.  **Spleen:** moderate, diffuse, congestion; mild, multifocal, red pulp infiltration of Mast cells.  **Heart, pancreas, skin, tongue, and thyroid:** NSFO. |
| MM395 | Thickening of the right mediastinal pleura and left ventricle; dark spots in the liver. | **Kidney:** marked, focally extensive, subcapsular acute hemorrhage.  **Skin:** mild, multifocal, keratinocyte individualized necrosis/apoptosis.  **Lungs:** alveolar edema; moderate autolysis.  **Heart, testicle:** NSFO.  **Liver, lymph node, pancreas, skeletal muscle:** advanced autolysis. |
| MM405 | Skin lesions compatible with gillnet entanglement in the rostrum, pectoral, dorsal and caudal fins; moderate meningeal congestion; pulmonary congestion; focal endocardiosis of the mitral valve. | **Adrenal gland:** mild, multifocal, cortico-medullar congestion.  **Kidney:** mild, multifocal, corticomedullar congestion; mild, multifocal tubular mineralization.  **Liver:** mild, multifocal, sinusoidal congestion; mild, multifocal, hepatocellular single cell necrosis/apoptosis; mild, multifocal, hydropic degeneration of hepatocytes; mild, focal, mixed (lymphocytic, neutrophilic) portal infiltrates; mild, focal, granulomatous hepatitis; mild, multifocal, ductular reaction; mild, multifocal, sinusoidal infiltrates of Mast Cells.  **Lung:** moderate, subpleural edema.  **Lymph node:** mild to moderate, multifocal, histiocytic lymphadenitis; mild, multifocal, sinus infiltrates of Mast Cells.  **Heart, pancreas, skin, thymus, tongue, trachea, uterus:** NSFO.  **Intestine:** advanced autolysis. |
| MM420 | Moderate axial and appendicular muscle atrophy; presence of vibrissae hair, skin lesions compatible with gillnet entanglement in the rostrum and peduncle; marked meningeal congestion; fatty liver. | **Liver:** moderate, multifocal, macrogoticular steatosis.  **Lungs:** mild, multifocal, alveolar histiocytosis; mild, multifocal, acute alveolar hemorrhage.  **Cerebrum, cerebellum, skin:** NSFO. |
| MM421 | Marked axial and appendicular muscle atrophy; moderate blubber depletion; fragment of gillnet in the rostrum; pulmonary congestion; moderate gastritis; focal pale firm punctual lesion in the hepatic capsule. | **Kidney:** mild, multifocal, corticomedullar congestion.  **Liver:** moderate, focally extensive, dissociation of hepatic trabecula; mild, multifocal, sinusoidal infiltrates of Mast Cells; mild, multifocal, hepatocellular single cell necrosis/apoptosis; mild, multifocal, hydropic degeneration of hepatocytes.  **Lungs:** moderate, focally extensive, septal congestion.  **Heart, ovary, pancreas, small intestine, stomach, thymus:** NSFO. |
| MM548 | Skin lesions compatible with gillnet entanglement; pulmonary edema; nodular lesions in the pulmonary parenchyma. | **Intestine:** moderate, multifocal, MALT follicular hyperplasia.  **Lung:** mild, focally extensive acute alveolar hemorrhage; edema and congestion; advanced autolysis.  **Lymph node:** moderate, multifocal, follicular hyperplasia.  **Skeletal muscle:** mild/moderate, multifocal, myofiber degeneration and necrosis.  **Skin:** moderate, multifocal, keratinocyte ballooning degeneration; mild, multifocal acanthosis; mild, multifocal mononuclear (lymphocytic, histiocytic) infiltrate in dermis.  **Aorta, diaphragm, eye, heart, liver, small intestine, spleen, thymus, trachea, urinary bladder:** NSFO.  **Adrenal gland, cerebellum, cerebrum, kidney, stomach, spinal cord, testicle:** advanced autolysis. |
| MM549 | Brownish circular erosions in the skin of 2 cm in diameter, at the end of the right pectoral fin; skin lesions compatible with gillnet entanglement in the pectoral fins; complete fracture of maxillary and mandibular bones; bruise and edema above the left scapula; multiorgan congestion; presence of two puncture lesions in the right lung and two in the left lung; 40 ml of serous sanguinolent liquid in the abdominal cavity; 3 mm in diameter whitish focal lesions in the left lobule of the parietal liver area. | **Skin:** mild/moderate, multifocal, keratinocyte ballooning degeneration; moderate autolysis. |
| MM550 | Mild axial and appendicular muscle atrophy; skin lesions compatible with gillnet entanglement in the pectoral and caudal fins; pulmonary edema; multifocal pale punctual lesions in lung parenchyma; hepatic congestion; lymphadenomegaly and splenomegaly. | **Large artery:** marked, focally extensive, serosal hemorrhage.  **Lung:** moderate, multifocal to coalescent, mixed (neutrophilic, histiocytic) bronchointerstitial pneumonia with multinucleate giant cells; moderate, multifocal, alveolar histiocytosis and foamy macrophages; moderate, multifocal, alveolar edema.  **Large intestine:** mild to moderate, multifocal, MALT follicular hyperplasia.  **Lymph node:** moderate, multifocal, follicular hyperplasia.  **Skeletal muscle:** mild, multifocal, myofiber degeneration and necrosis.  **Skin:** mild, multifocal, keratinocyte ballooning degeneration.  **Cerebrum, diaphragm, eye, heart, liver, skin, spleen, urinary bladder:** NSFO.  **Adrenal gland, stomach, small intestine, kidney, thymus:** advanced autolysis. |
| MM566 | Skin lesions compatible with gillnet entanglement in the dorsal and pectoral fins; presence of few multifocal whitish caseous lesions ranging from 0.3 to 0.6 cm in liver and lungs. | **Cerebrum:** mild, multifocal, hemorrhage of the neuroparenchyma; mild, multifocal, congestion.  **Cerebellum:** mild, multifocal, hemorrhage of the neuroparenchyma.  **Intestine:** mild/moderate, focal, mixed (eosinophilic, histiocytic) enteritis with intralesional metazoan larvae (nematode); mild, multifocal, congestion.  **Kidney:** mild/moderate, multifocal, corticomedullar congestion.  **Liver:** mild, multifocal, hepatocellular single cell necrosis/apoptosis; mild, multifocal, hydropic degeneration of hepatocytes.  **Lung:** marked, diffuse, alveolar edema and congestion; mild to moderate, multifocal, alveolar histiocytosis.  **Lymph node:** mild, multifocal, sinus infiltrates of Mast Cells; mild, multifocal, follicular hyperplasia.  **Skeletal muscle:** mild, multifocal, myofiber degeneration and necrosis.  **Skin:** mild, multifocal, keratinocyte ballooning degeneration.  **Spleen:** mild/moderate, multifocal, histiocytosis in red pulp.  **Adrenal gland, heart, pancreas, testicle, tongue:** NSFO. |
| MM568 | Presence of vibrissae hair; skin lesions compatible with gillnet entanglement in the rostrum, dorsal and pectoral fins and dorsum; whitish to greyish well circumscribed skin lesion in the left ventral side of the caudal fin up to 9 cm in length; pulmonary congestion; focal grayish areas in the spleen surface; pale areas in the liver. | **Adrenal gland:** mild, focal, acute perivascular hemorrhage.  **Kidney:** mild/moderate, multifocal, corticomedullar congestion.  **Liver:** mild, multifocal, hepatocellular single cell necrosis/apoptosis; mild, multifocal, hydropic degeneration of hepatocytes.  **Lung:** mild, multifocal, perivascular edema; mild, multifocal, subpleural edema; mild, multifocal, alveolar histiocytosis.  **Lymph node:** mild, multifocal, granulomatous lymphadenitis with rare intranuclear eosinophilic inclusion bodies.  **Skeletal muscle:** mild, multifocal, myofiber degeneration.  **Skin:** mild, focal, acute internal dermatitis.  **Aorta, cerebrum, eye, heart, pancreas, skeletal muscle, small intestine, tongue, thymus:** NSFO. |
| ii151268 | Presence of vibrissae hair, umbilical cord and fetal folds; marked, generalized multi-organ congestion; marked, diffused pulmonary edema; moderate hydropericardium (5 ml); mild to moderate, diffuse hyperemia of gastric mucosa associated with absence of food/milk. | **Liver**: mild, multifocal, hepatocellular single cell necrosis/apoptosis.  **Lung**: Mild/moderate, multifocal, alveolar edema; mild, multifocal, alveolar histiocytosis.  **Skeletal muscle**: mild, multifocal, myofiber degeneration and necrosis.  **Bone narrow, eye, heart, lymph node, skin. small intestine, thymus, thyroid, uterus**: NSFO. |
| ii166901 | Moderate axial and appendicular muscle atrophy; moderate blubber depletion; linear cutaneous laceration and imprints in rostrum, peduncle and pectoral and dorsal fins, suggestive of gillnet entanglement; moderate, multifocal, subcutaneous hemorrhage, contusion and edema; moderate, generalized organ congestion; mild, diffuse hyperemia of cervical mucosa; mild presence of plastic debris on stomach. | **Adrenal gland**: minimal, focal, mixed (lymphocytic, neutrophilic) adrenalitis.  **Cerebrum**: mild, multifocal, gliosis.  **Heart**: mild, multifocal, cardiomyocyte degeneration and necrosis.  **Intestine**: mild, multifocal, MALT hyperplasia, minimal, mononuclear (lymphocytic) enteritis.  **Liver**: mild/moderate, multifocal, sinusoidal congestion; mild, multifocal, midzonal hemorrhage; mild, multifocal, ductular reaction; minimal, focal, acute (neutrophilic) lobular hepatitis.  **Lung**: mild, multifocal, alveolar histiocytosis; alveolar edema; moderate autolysis with putrefaction bacterial colonies.  **Lymph node**: mild/moderate, multifocal eosinophilic lymphadenitis; mild, multifocal, follicular hyperplasia.  **Skeletal muscle**: mild, multifocal, myofiber degeneration and necrosis.  **Skin**: mild, multifocal, keratinocyte ballooning degeneration.  **Thymus**: mild, multifocal, granulomatous thymitis; moderate autolysis with intravascular bacterial colonies.  **Kidney, intestine, thyroid**: NSFO. |
| ii169562 | Moderate axial and appendicular muscle atrophy; moderate, multifocal, not delineated, circular skin lesions in ventral region; moderate, multifocal, proliferative dermatitis in rostrum; moderate, multifocal to coalescent pale granular nodules in right lung parenchyma; mild to moderate, focal fibrosis in left lung; mild presence of foamy content on trachea associated with marked congestion; enlarged liver with cystic formation of approximately 6.2 x 5.0 x 3.2 cm, containing 52 ml of yellowish translucid liquid in ventral region of right hepatic lobe; marked, diffuse hyperemia of gastric mucosa with mild multifocal petechiae; focal renal cyst of 0,2 cm with dark yellow content; focal heart cyst of 0.5 cm in apical region of left ventricle; presence of a mummified fetus with associated marked, diffuse necrosis in uterine mucosa; marked brain congestion and mild multifocal hemorrhage. | **Heart:** mild, multifocal, congestion.  **Large intestine:** minimal, focal, mononuclear (lymphocytic, histiocytic) colitis.  **Liver:** mild, multifocal, ductular reaction; mild, multifocal, sinusoidal congestion.  **Skin:** mild, multifocal, keratinocyte ballooning degeneration.; mild, focal, acanthosis.  **Stomach:** minimal, focal, lymphocytic gastritis.  **Cerebrum:** NSFO. |

*NSFO: no significant findings observed
